# Distribution of Fimbrial Genes and Their Association with Virulence and Levofloxacin Resistance/Extended-Spectrum Beta-Lactamase Production in Uropathogenic *Escherichia coli*

**DOI:** 10.3390/antibiotics14050468

**Published:** 2025-05-06

**Authors:** Masao Mitsui, Takanori Sekito, Mai Maruhashi, Yuki Maruyama, Takehiro Iwata, Yusuke Tominaga, Satoshi Katayama, Shingo Nishimura, Kensuke Bekku, Motoo Araki, Hidetada Hirakawa, Takuya Sadahira

**Affiliations:** 1 Department of Urology, Okayama University Graduate School of Medicine, Dentistry and Pharmaceutical Sciences, 2-5-1, Shikata-cho, Kita-ku, Okayama 700-8558, Japan; me422095@s.okayama-u.ac.jp (M.M.); t.sekito410@gmail.com (T.S.); eat.the.lobster@gmail.com (Y.M.); pfty8bwj@s.okayama-u.ac.jp (T.I.); p3uq1s4o@s.okayama-u.ac.jp (Y.T.); pzgr6t3w@s.okayama-u.ac.jp (S.K.); pm5n8mce@s.okayama-u.ac.jp (S.N.); gmd421030@s.okayama-u.ac.jp (K.B.); motoosh@md.okayama-u.ac.jp (M.A.); 2 Department of Bacteriology, Graduate School of Medicine, Gunma University, 3-39-22, Showa-machi, Maebashi Gunma 371-8511, Japan; maruhashi@gunma-u.ac.jp (M.M.); hirakawa@gunma-u.ac.jp (H.H.); 3 Center for Innovative Clinical Medicine, Okayama University Graduate School of Medicine, Dentistry and Pharmaceutical Sciences, 2-5-1, Shikata-cho, Kita-ku, Okayama 700-8558, Japan

**Keywords:** fimbriae, urinary tract infection, drug resistance, virulence, uropathogenic *Escherichia coli*

## Abstract

**Background:** Urinary tract infection (UTI) is predominantly caused by uropathogenic *Escherichia coli* (UPEC). Previous studies have reported that the fimbriae of UPEC are involved in virulence and antimicrobial resistance. We aimed to analyze the fimbrial gene profiles of UPEC and investigate the specificity of these expressions in symptomatic UTI, urinary device use, and levofloxacin (LVFX) resistance/extended-spectrum beta-lactamase (ESBL) production. **Methods:** A total of 120 UPEC strains were isolated by urine culture between 2019 and 2023 at our institution. They were subjected to an antimicrobial susceptibility test and polymerase chain reaction (PCR) to identify 14 fimbrial genes and their association with clinical outcomes or antimicrobial resistance. **Results:** The prevalence of the papG2 gene was significantly higher in the symptomatic UTI group by multivariate analyses (OR 5.850, 95% CI 1.390–24.70, *p* = 0.016). The prevalence of the c2395 gene tended to be lower in the symptomatic UTI group with urinary devices (all *p* < 0.05). In LVFX-resistant UPEC strains from both the asymptomatic bacteriuria (ABU) and the symptomatic UTI group, the expression of the *papEF*, *papG3*, *c2395*, and *yadN* genes tended to be lower (all *p* < 0.05). **Conclusion:** The fimbrial genes of UPEC are associated with virulence and LVFX resistance, suggesting that even UPEC with fewer motility factors may be more likely to ascend the urinary tract in the presence of the urinary devices. These findings may enhance not only the understanding of the virulence of UPEC but also the management of UTI.

## 1. Introduction

Urinary tract infection (UTI) is among the most common bacterial infections encountered in clinical practice, predominantly caused by uropathogenic *Escherichia coli* (UPEC) [1,2]. UTI can be seen in a wide spectrum ranging from asymptomatic bacteriuria (ABU) to pyelonephritis and urosepsis, resulting in increased morbidity and medical costs [3,4,5]. Numerous virulence factors of UPEC have been determined [6,7,8,9], particularly fimbriae as an adherence factor, playing a role in colonizing the urinary tract [10,11,12]. The ability of *E. coli* to adhere to the uroepithelial cells is a critical virulence factor, and this adherence is largely mediated by fimbriae, particularly type 1 fimbriae and P fimbriae [13,14,15,16]. Previous studies showed that P fimbriae are prevalent among UPEC strains associated with pyelonephritis [17]. This finding indicates an association between fimbriae and UTI. Additionally, the use of urinary device has been reported to increase the incidence of UTI in numerous studies [5,18,19,20]. Although there have been a few reports on the relationship between type 1 fimbriae and urinary devices [21], comprehensive analyses of the relationship between fimbriae, virulence, and urinary devices have been limited, and this relationship remains unclear. Furthermore, the increasing prevalence of antimicrobial-resistant bacteria, including levofloxacin (LVFX)-resistant and extended-spectrum β-lactamase (ESBL)-producing UPEC strains, has emerged as a significant concern in the management of UTI [22,23,24]. While the association between fimbriae and levofloxacin resistance/extended-spectrum beta-lactamase production has been reported [25,26,27,28], few studies have evaluated antimicrobial resistance based on virulence and fimbriae [29]. Understanding the fimbrial genes associated with virulence is essential for developing effective treatment strategies and managing the increasing incidence of UTI caused by antimicrobial-resistant bacteria and the use of urinary devices.

In this study, we aimed to analyze the fimbrial gene profiles of UPEC and investigate the specificity of these expressions in the symptomatic UTI group, the use of urinary devices, and antimicrobial-resistant strains. By investigating the specificity, we aim to better understand how these genes relate to virulence and antimicrobial resistance.

## 2. Results

### 2.1. Patient Demographics and Distribution of Fimbrial Genes

The ABU group consisted of 39 women (56.5%) and 30 men (43.5%), with an average age of 64 years, including 15 patients with diabetes (21.7%). The symptomatic UTI group consisted of 32 women (62.7%) and 19 men (37.3%), with an average age of 65.8 years, including 13 patients with diabetes (25.5%). A total of 120 UPEC strains were analyzed using PCR to assess the prevalence of 14 fimbrial genes, including *fimH*, *papEF*, *papG1*, *papG2*, *papG3*, *sfaS*, *focG*, *afa/draBC*, *bmaE*, *gafD*, *c2395*, *ppdD*, *yadN*, and *ygiL* (Table 1). The *fimH* and *ppdD* genes were the most prevalent and were present in all strains (120/120). The other genes, listed in order of prevalence, were as follows: *ygiL* (71.7%), *yadN* (32.5%), *c2395* (26.7%), *papEF* (22.5%), *papG3* (15%), *papG2* (10.8%), *papG1* (10%), *sfaS* (6.7%), *focG* (5%), *afa/draBC* (2.5%), *bmaE* (0%), and *gafD* (0%).

### 2.2. Comparison of the Distribution of Fimbrial Genes Between ABU and Symptomatic UTI Groups

All UPEC strains were divided into ABU (n = 69) and symptomatic UTI (n = 51) groups (Table 2). The *papG2* gene was significantly more prevalent in the symptomatic UTI group, with a prevalence of 19.6% (10/51), compared to 5.8% (4/69) in the ABU group (*p* = 0.024). Multivariate analysis showed that *papG2* was significantly associated with symptomatic UTI (OR 5.850, 95% CI 1.390–24.70, *p* = 0.016) (Table 3). There were no significant differences in the distribution of other fimbrial genes between the two groups (all *p* > 0.05). Both groups were further classified by urinary device use. The prevalence of the use of urinary devices was 49% (34/69) in the ABU group and 51% (26/51) in the symptomatic UTI group (Table 4). The prevalence of the *papG2* gene was significantly higher in the symptomatic UTI group without a urinary device than with a urinary device (*p* = 0.036), and the prevalence of the *c2395* gene was significantly lower in the symptomatic UTI group with a urinary device than without a urinary device (*p* = 0.013).

### 2.3. Comparison of the Distribution of Fimbrial Genes by Antimicrobial Susceptibility Testing

Both groups were further classified by antimicrobial susceptibility. LVFX-resistant strains were 57% prevalent (39/69) in the ABU group and 61% (31/51) in the symptomatic UTI group (Table 5). In LVFX-resistant UPEC, a decrease in *papEF*, *papG3*, *c2395*, and *yadN* was suggested in both the ABU group and the symptomatic UTI group compared to LVFX-susceptible UPEC (all *p* < 0.05). Additionally, a decrease in sfaS was suggested in the LVFX-resistant UPEC of the symptomatic UTI group. The prevalence of ESBL-producing strains was 38%, with 69% (26/69) in the ABU group and 39% (20/51) in the symptomatic UTI group (Table 6). In ESBL-producing UPEC, a decrease in *papEF*, *focG*, *c2395*, and *yadN* was suggested in both the ABU group and the symptomatic UTI group compared to non-ESBL-producing UPEC (all *p* < 0.05). No significant differences were observed in the distribution of other fimbrial genes (all *p* > 0.05). All strains were classified into four groups: 48 non-ESBL-producing group and LVFX-susceptible, 26 non-ESBL-producing group and LVFX-resistant (including -intermediate) group, 2 ESBL-producing group and LVFX-susceptible group, and 44 ESBL-producing group and LVFX-resistant (including -intermediate) group (Table 7). We compared the fimbrial genes in ESBL production under the same LVFX conditions, and *focG* was significantly lower in ESBL-producing strains (*p* = 0.048). No significant differences were observed in the distribution of the other fimbrial genes (all *p* > 0.05).

## 3. Discussion

The present study provides virulence and antimicrobial resistance associated with fimbriae in UPEC strains. We have identified significant differences in the expression of specific genes by analyzing the prevalence of the fimbrial genes in UPEC strains from both the ABU and the symptomatic UTI groups. The *papG2* gene was significantly more prevalent in the symptomatic UTI, and it was suggested that *papG2* may be related to virulence. Furthermore, in the symptomatic UTI group with a urinary device, the expression rate of the c2395 gene, which is suggested to be associated with motility, was significantly lower compared to the symptomatic UTI group without a urinary device. This finding suggests that such devices may allow bacteria with limited fimbriae involved in motility to ascend the urinary tract, and the UPEC potentially exhibit virulence. In addition, comparative analysis between LVFX-sensitive and -resistant strains suggested that the expression of fimbrial genes such as papEF, papG3, sfaS, c2395, and yadN was decreased in LVFX-resistant strains. These findings not only improve our understanding of the virulence of UPEC but may also help manage UTI to solve increasing antimicrobial-resistant bacteria and medical costs.

Our study further supports existing data that has previously linked fimbrial genes to the virulence of UPEC. In a previous study, various types of fimbrial genes have been analyzed as virulence factors. FimH, necessary for adhesion to the bladder epithelium, is highly expressed in 95–100% of UPEC [7,25,30], suggesting that it is essential for colonization of the urinary tract. Although there are few reports on PpdD, it has been suggested to be important for urothelial adhesion and has been reported to be highly expressed in 97–100% of UPEC isolated from humans and animals [31,32]. The *papG2* has been associated with pyelonephritis and upper urinary tract infections in numerous studies [8,31,33,34,35]. In our study, *fimH* and *ppdD* were expressed in all UPEC strains (100%), and *papG2* was significantly elevated in the symptomatic UTI group (OR 5.850, 95% CI 1.390–24.70, *p* = 0.016), supporting previous studies. Adhesive fimbria, such as type 1 and P-fimbria, contain adhesins at the tips that play important roles in UTI [36,37,38]. FimH, the type 1 fimbria adhesin, binds to the mannosylated glycoproteins on the surface of human bladder epithelial cells and promotes facilitating bacterial colonization, invasion, and biofilm formation [39,40]. This factor is essential for the effective colonization of the urinary tract and is consequently a frequently identified fimbrial virulence factor of UPEC in our data. PapG, the P-fimbria adhesin, exists in three alleles (PapG1, G2, and G3). Each PapG allele is known to have a distinct isoreceptor specificity, which, in turn, results in altered host tissue tropism [41]. PapG2 binds to the Gala1–4Gal (galabiose) on the surface of human kidney epithelial cells, is involved in kidney colonization [42,43,44], and consequently establishes a robust inflammatory response during renal colonization [34]. Our data also indicated that this factor was significantly associated with symptomatic UTI, consistent with previous reports. However, not all UPEC strains associated with pyelonephritis possess *papG2*, indicating that other virulence factors may contribute to symptomatic UTI. In the symptomatic UTI group without a urinary device, the *papG2* gene was significantly more prevalent than in the symptomatic UTI group with a urinary device. The use of urinary devices has been reported to increase the incidence of UTI, which is believed to be due to the adhesion of bacteria and the formation of biofilms [22,23,24,45]. Although there have been a few reports on its relationship with type 1 fimbriae [24,46], all UPEC strains possessed type 1 fimbriae, so our study had no specificity. These findings suggest that *papG2* may possess virulence and an enhanced ability to ascend the urinary tract without relying on urinary devices. The finding that *c2395*, which is suggested to be associated with motility [47], was significantly lower in the group with symptomatic urinary tract infections who had urinary tract devices suggests that such a device may facilitate the ascent of UPEC with limited motility, potentially enhancing its pathogenicity.

Regarding antibiotic resistance, this study further supports existing data that demonstrate a correlation between UPEC and fimbrial genes. Quinolone-resistant UPEC had lower fimbrial genes than quinolone-susceptible UPEC in numerous studies [26,27,28]. ESBL-producing strains were previously found to have lower fimbrial genes than non-ESBL-producing ones [25,48,49,50], but there were differences in the type of fimbrial genes among the reports, and no consensus has been reached. In our study, fimbrial genes such as *papEF*, *papG3*, *sfaS*, *c2395*, and *yadN* were suggested to be less abundant in LVFX-resistant UPEC (all *p* < 0.05). This data showed that LVFX-resistant UPEC tends to have lower fimbrial genes, supporting previous studies. Similar results were observed with ESBL-producing strains, but only two strains (4.3%) were LVFX-susceptible UPEC among them, suggesting potential confounding effects due to LVFX-resistant UPEC. We compared the fimbrial genes in ESBL production under the same LVFX conditions; the *focG* genes were significantly lower (*p* < 0.05), and there were no significant differences in the other fimbrial genes (all *p* > 0.05) (Table 7). These results suggested a weak relationship between ESBL-producing bacteria and fimbrial genes. However, a consensus has yet to be established, and further analysis involving a larger number of strains is warranted.

In quinolone-resistant UPEC, two hypotheses have been proposed to explain the reduced virulence factors. The first hypothesis is that quinolone-resistant bacteria and the reduction of fimbrial genes are associated with the phylogenetic origins of *E. coli*, as demonstrated in several studies [51,52]. These genotypes of quinolone-resistant bacteria more closely resemble the phylogenetic characteristics and virulence factor profiles of animal-isolated strains than human quinolone-susceptible bacteria. This finding may have resulted from foodborne infections linked to the excessive use of fluoroquinolones in food animal production [52,53]. However, genetic diversity has been reported among quinolone-resistant bacteria, leading to arguments against this hypothesis [28]. The second hypothesis is that there may be a relationship between antimicrobial resistance genes and fimbrial genes. Antimicrobial resistance may primarily arise from mutations in target genes [54] or through the acquisition of resistance genes via mobile genetic elements such as plasmids and integrons [55]. A previous study showed that horizontal transmission of antimicrobial resistance genes leads to the downregulation of fimbrial genes [56]. This finding may play a role in suppressing the expression of fimbrial genes, thereby protecting the pathogen from attacks and detection by the host’s immune response. The relationship between drug resistance and fimbrial genes has not yet been clarified, and further research is needed to investigate these hypotheses.

The implications of our findings are multifaceted. The identification of *papG2* as a significant virulence factor in symptomatic UTI cases suggests that it may serve as a biomarker for assessing the risk of symptomatic UTI. Moreover, in cases where urinary devices are used, even if there are few motility factors, it may be better to treat them proactively if there are virulence factors. Administering antimicrobials selectively to cases with virulence factors may reduce the unnecessary use of antimicrobial agents and consequently lower medical costs. Furthermore, numerous reports have indicated that polyvalent mannose-based FimH inhibitors exhibit antiadhesive effects against UPEC [57,58,59]. Understanding the mechanisms of fimbrial virulence factors may make it possible to develop new therapeutic strategies to inhibit bacterial adhesion and thereby prevent infections. In addition, identifying the reduced expression of fimbrial genes in quinolone-resistant UPEC may provide new insights regarding the use of antimicrobial agents. Specifically, this information could serve as a basis for determining which antimicrobial agents to select when an infection with resistant strains is suspected. To further evaluate the relationship between fimbrial genes and virulence, as well as antimicrobial resistance, studies utilizing gene knockout strains and comprehensive research that includes genes such as gyrA [60], which have been implicated in pathogenicity and antimicrobial resistance, are necessary.

Despite the significant findings, our study has several limitations. First, UPEC was evaluated at a single institution, resulting in a limited number of cases (such as LVFX-susceptible/ESBL-producing strains). Second, individual urinary tract infections, such as cystitis and pyelonephritis, were analyzed within the same group as symptomatic UTIs. Third, bacterial pathogenicity is often multifactorial and may depend on specific combinations or patterns of virulence genes rather than isolated factors. Finally, our analysis did not consider other elements of virulence in UPEC, such as toxins, iron acquisition systems, and immune evasion strategies.

## 4. Materials and Methods

### 4.1. Study Design and Bacterial Strains

We analyzed a total of 120 UPEC strains isolated from urine cultures and preserved at Okayama University Hospital from 2019 to 2023. All strains collected during the study period were included, and no exclusion criteria were applied. We collected medical records and clinical data from medical records, classified UPEC strains into the asymptomatic bacteriuria (ABU) group and the symptomatic UTI group, and then analyzed the fimbrial genes. In addition, we analyzed the genes by classifying the strains into the use of urinary devices, LVFX-susceptible or -resistant, and ESBL-producing or non-ESBL-producing strains. We defined ABU as the detection of bacteria in urine culture at a concentration of ≥10^5^ colony-forming units (CFUs)/mL without the symptoms of a UTI. The symptomatic UTI group includes acute pyelonephritis, acute cystitis, and acute prostatitis. Acute pyelonephritis was clinically defined as a disease accompanied by fever (armpit temperature > 37.5 °C), lumbar tenderness, and pyuria. Acute cystitis is defined as a disease accompanied by significant bacteriuria, which is associated with inflammation of the bladder mucosa without fever. The urinary device group includes urinary catheters, ureteral stents, and nephrostomy catheters.

### 4.2. Culture Conditions

Isolated UPEC strains were labeled with batch numbers, cultured on Casitone agar and stored at room temperature. When used for analysis, the strains were transferred to standard agar plates for further cultivation. After being cultivated on slant agar prepared in test tubes, UPEC was preserved under refrigeration and shipped to Gunma University. After arriving at Gunma University, UPEC was cultured overnight at 37 °C in Luria–Bertani medium without shaking.

### 4.3. Detection of Fimbrial Genes

The genes of 14 known fimbriae of UPEC were detected by the polymerase chain reaction (PCR), including the *fimH* (type 1 fimbriae), *papEF* (P fimbriae), *papG* class I to III alleles (PapG adhesins of P fimbriae), *sfaS* (S fimbriae), *focG* (F1C fimbriae), *afa/draBC* (Afa/Dr fimbriae), *bmaE* (M fimbriae), *gafD* (G fimbriae), *c2395* (Putative type IV pili), *ppdD* (Putative type IV pili), *yadN* (Yad fimbriae), and *YgiL* (Ygi fimbriae). The PCR primers for the genes are presented in Table 8. The primers used in this study were validated based on previous literature [7,31,46,61,62,63].

### 4.4. DNA Extraction and Real-Time PCR Analysis

We harvested bacterial cells from 0.4 mL of an overnight culture pellet, and the boiling method (98 °C, 10 min) extracted the DNA from the isolates. Amplification was done in a 25 μL reaction mixture containing template DNA (2 μL of boiled lysate). We attempted PCR analysis with Quick Taq^TM^ HS DyeMix (Toyobo, Osaka, Japan). The following PCR conditions were used: For *papG* class I to III alleles, initial denaturation at 94 °C for 5 min, followed by 25 cycles of denaturation at 94 °C for 30 s, annealing at 55 °C for 30 s, and extension at 68 °C for 3 min, with a final extension at 72 °C for 10 min. For the other fimbrial genes, initial denaturation at 94 °C for 5 min, followed by 25 cycles of denaturation at 94 °C for 30 s, annealing at 63 °C for 30 s, and extension at 68 °C for 3 min, with a final extension at 72 °C for 10 min. Finally, all the PCR products were electrophoresed on a 3% agarose gel and stained with ethidium bromide, then visualized under UV light.

### 4.5. Antimicrobial Susceptibility Testing

Antimicrobial susceptibility testing was conducted using the standard disc diffusion technique according to the Clinical and Laboratory Standards Institute (CLSI) guidelines [64]. The samples were cultured on bromothymol blue (BTB) agar medium. After 16–20 h of aerobic incubation at 35 °C, the susceptibility results were interpreted based on the MIC breakpoints of LVFX: susceptible at MIC ≤ 0.5, intermediate at MIC of 1, and resistance at MIC ≥ 2 μg/mL for *E*. *coli* according to CLSI VET01S [65]. For analysis, LVFX-intermediate was included in LVFX-resistance as insusceptibility. ESBL production was investigated using ceftazidime/clavulanic acid and cefotaxime/clavulanic acid discs (30/10 μg) alongside cefpodoxime/clavulanic discs (10/10 μg).

### 4.6. Statistical Analysis

For statistical analysis, Fisher’s exact test was performed to evaluate differences between the ABU group and symptomatic UTI groups. Multivariable logistic regression was performed to estimate the odds of being in the symptomatic UTI group (vs. ABU group), based on the presence of five virulence genes: *papEF*, *papG2*, *papG3*, *sfaS*, and *afa/draBC*. We compared the use of urinary devices or antimicrobial-resistant bacteria between the two groups using Fisher’s exact test, with *p* < 0.05 indicating statistical significance. All analyses were conducted using EZR software at Saitama Medical Center, Jichi Medical University.

## 5. Conclusions

The present study showed that the *papG2* gene was a virulence factor. It was suggested that the *papG2* gene has an excellent ability to ascend the urinary tract, regardless of the presence or absence of a urinary device. Our findings associated with the *c2395* gene suggested that UPEC may be potentially pathogenic, because the urinary tract device allows bacteria with limited motility to ascend the urinary tract. In addition, the number of fimbrial genes was significantly lower in LVFX-resistant strains, which may lead to the early detection of antimicrobial-resistant bacteria. These findings not only improve our understanding of the virulence of UPEC but may also assist in the management of UTI.

## Figures and Tables

**Table 1:** Distribution of fimbrial genes among 120 *E. coli* strains.

Gene	Description	Total (n = 120)
*fimH*	Type 1 fimbria adhensin	120 (100%)
*papEF*	P fimbria	27 (22.5%)
*papG1*	P fimbria adhesin (allele 1)	12 (10%)
*papG2*	P fimbria adhesin (allele 2)	13 (10.8%)
*papG3*	P fimbria adhesin (allele 3)	18 (15%)
*sfaS*	S fimbria	8 (6.7%)
*focG*	F1c fimbria	6 (5%)
*afa/draBC*	Afa/Dr fimbriae	3 (2.5%)
*bmaE*	M fimbriae	0 (0.0%)
*gafD*	G fimbria	0 (0.0%)
*c2395*	Putative type IV pili	32 (26.7%)
*ppdD*	Putative type IV pili	120 (100%)
*yadN*	Yad fimbriae	39 (32.5%)
*ygiL*	Ygi fimbriae	86 (71.7%)

**Table 2:** Differences in fimbrial genes between the ABU group and symptomatic UTI group.

Gene	ABU (n = 69)	Symptomatic UTI (n = 51)	*p* Value
*fimH*	69 (100%)	51 (100%)	
*papEF*	15 (21.7%)	12 (23.5%)	0.828
*papG1*	9 (13%)	4 (7.8%)	0.554
*papG2*	4 (5.8%)	10 (19.6%)	0.024
*papG3*	10 (14.5%)	9 (17.6%)	0.801
*sfaS*	5 (7.2%)	4 (7.8%)	ns
*focG*	4 (5.8%)	3 (5.9%)	ns
*afa/draBC*	2 (2.9%)	2 (3.9%)	ns
*bmaE*	0 (0.0%)	0 (0.0%)	
*gafD*	0 (0.0%)	0 (0.0%)	
*c2395*	19 (27.5%)	14 (27.5%)	ns
*ppdD*	69 (100%)	51 (100%)	
*yadN*	24 (34.8%)	16 (31.4)	0.845
*ygiL*	51 (73.9%)	36 (70.6%)	0.686

ABU, asymptomatic bacteriuria; Symptomatic UTI, symptomatic urinary tract infection; ns, not significant.

**Table 3:** Multivariate logistic regression analysis of the risk factors for symptomatic UTI.

Gene	Odds Ratio	(95% CI)	*p* Value
*papEF*	0.385	(0.075, 1.970)	0.251
*papG2*	5.850	(1.390, 24.70)	0.016
*papG3*	3.300	(0.481, 22.60)	0.224
*sfaS*	1.020	(0.161, 6.500)	0.980
*afa/draBC*	1.090	(0.128, 9.230)	0.938

CI, confidence interval.

**Table 4:** Distribution of fimbrial genes with and without a urinary device.

Gene	ABU (n = 69)		*p* Value	Symptomatic UTI (n = 51)		*p* Value
	Device^−^ (n = 35)	Device^+^ (n = 34)		Device^−^ (n = 25)	Device^+^ (n = 26)	
*fimH*	35 (100%)	34 (100%)		25 (100%)	26 (100%)	
*papEF*	8 (22.9%)	7 (20.6%)	ns	8 (32.0%)	4 (15.4%)	0.199
*papG1*	7 (20.0%)	2 (5.9%)	0.151	2 (8.0%)	2 (7.7%)	ns
*papG2*	2 (5.7%)	2 (5.9%)	ns	8 (32.0%)	2 (7.7%)	0.036
*papG3*	5 (14.3%)	5 (14.7%)	ns	4 (16.0%)	5 (19.2%)	ns
*sfaS*	3 (8.6%)	2 (5.9%)	ns	2 (8.0%)	2 (7.7%)	ns
*focG*	2 (5.7%)	2 (5.9%)	ns	3 (12.0%)	0 (0.0%)	0.110
*afa/draBC*	1 (2.9%)	1 (2.9%)	ns	1 (4.0%)	1 (3.8%)	ns
*bmaE*	0 (0.0%)	0 (0.0%)		0 (0.0%)	0 (0.0%)	
*gafD*	0 (0.0%)	0 (0.0%)		0 (0.0%)	0 (0.0%)	
*c2395*	10 (28.6%)	9 (26.5%)	ns	11 (44.0%)	3 (11.5%)	0.013
*ppdD*	35 (100%)	34 (100%)		25 (100%)	26 (100%)	
*yadN*	10 (28.6%)	14 (41.2%)	0.318	10 (40.0%)	6 (23.1%)	0.237
*ygiL*	26 (74.3%)	25 (73.5%)	ns	19 (76.0%)	17 (65.4%)	0.541

ABU, asymptomatic bacteriuria; Symptomatic UTI, symptomatic urinary tract infection; ns, not significant.

**Table 5:** Differences of fimbrial genes among levofloxacin-susceptible and -resistant strains.

Gene	ABU (n = 69)		*p* Value	Symptomatic UTI (n = 51)		*p* Value
	LVFX^S^ (n = 30)	LVFX^R^ (n = 39)		LVFX^S^ (n = 20)	LVFX^R^ (n = 31)	
*fimH*	30 (100%)	39 (100%)		20 (100%)	31 (100%)	
*papEF*	11 (36.7%)	4 (10.3%)	0.017	11 (55.0%)	1 (3.2%)	<0.001
*papG1*	5 (16.7%)	4 (10.3%)	0.488	2 (10.0%)	2 (6.5%)	0.640
*papG2*	3 (10.0%)	1 (2.6%)	0.310	5 (25.0%)	5 (16.1%)	0.486
*papG3*	8 (26.7%)	2 (5.1%)	0.016	8 (40.0%)	1 (3.2%)	0.001
*sfaS*	4 (13.3%)	1 (2.6%)	0.159	4 (20.0%)	0 (0.0%)	0.019
*focG*	2 (6.7%)	2 (5.1%)	ns	2 (10.0%)	1 (3.2%)	0.553
*afa/draBC*	0 (0.0%)	2 (5.1%)	0.501	0 (0.0%)	2 (6.5%)	0.514
*bmaE*	0 (0.0%)	0 (0.0%)		0 (0.0%)	0 (0.0%)	
*gafD*	0 (0.0%)	0 (0.0%)		0 (0.0%)	0 (0.0%)	
*c2395*	14 (46.7%)	5 (12.8%)	0.003	10 (50.0%)	4 (12.9%)	0.009
*ppdD*	30 (100%)	39 (100%)		20 (100%)	31 (100%)	
*yadN*	18 (60.0%)	6 (15.4%)	<0.001	12 (60.0%)	4 (12.9%)	<0.001
*ygiL*	23 (76.7%)	28 (71.8%)	0.784	16 (80.0%)	20 (64.5%)	0.348

ABU, asymptomatic bacteriuria; Symptomatic UTI, symptomatic urinary tract infection; LVFX^S^, Levofloxacin-susceptible strains; LVFX^R^, Levofloxacin-resistant strains.

**Table 6:** Differences of fimbrial genes among ESBL-producing and non-producing strains.

Gene	ABU (n = 69)		*p* Value	Symptomatic UTI (n = 51)		*p* Value
	ESBL^−^ (n = 43)	ESBL^+^ (n = 26)		ESBL^−^ (n = 31)	ESBL^+^ (n = 20)	
*fimH*	43 (100%)	26 (100%)		31 (100%)	20 (100%)	
*papEF*	13 (30.2%)	2 (7.7%)	0.036	10 (32.3%)	2 (10.0%)	0.095
*papG1*	7 (16.3%)	2 (7.7%)	0.466	3 (9.7%)	1 (5.0%)	ns
*papG2*	4 (9.3%)	0 (0.0%)	0.289	7 (22.6%)	3 (15.0%)	0.721
*papG3*	9 (20.9%)	1 (3.8%)	0.077	9 (29.0%)	0 (0.0%)	0.008
*sfaS*	4 (9.3%)	1 (3.8%)	0.643	4 (12.9%)	0 (0.0%)	0.145
*focG*	4 (9.3%)	0 (0.0%)	0.289	3 (9.7%)	0 (0.0%)	0.271
*afa/draBC*	1 (2.3%)	1 (3.8%)	ns	2 (6.5%)	0 (0.0%)	0.514
*bmaE*	0 (0.0%)	0 (0.0%)		0 (0.0%)	0 (0.0%)	
*gafD*	0 (0.0%)	0 (0.0%)		0 (0.0%)	0 (0.0%)	
*c2395*	16 (37.2%)	3 (11.5%)	0.027	14 (45.2%)	0 (0.0%)	<0.001
*ppdD*	43 (100%)	26 (100%)		31 (100%)	20 (100%)	
*yadN*	21 (48.8%)	3 (11.5%)	0.002	15 (48.4%)	1 (5.0%)	0.001
*ygiL*	32 (74.4%)	19 (73.1%)	ns	21 (67.7%)	15 (75.0%)	0.755

ABU, asymptomatic bacteriuria; Symptomatic UTI, symptomatic urinary tract infection; ESBL^−^, extended-spectrum beta-lactamase-non-producing strains; ESBL^+^, extended-spectrum beta-lactamase-producing strains; ns, not significant.

**Table 7:** Distribution of fimbrial genes among levofloxacin-susceptible or -resistant strains and ESBL-producing or -non-producing strains.

Gene	ESBL^−^ LVFX^S^ (n = 48)	ESBL^−^ LVFX^R^ (n = 26)	ESBL^+^ LVFX^S^ (n = 2)	ESBL^+^ LVFX^R^ (n = 44)	ESBL^−^ LVFX^R^ vs. ESBL^+^ LVFX^R^ (*p* Value)
*fimH*	48 (100%)	26 (100%)	2 (100%)	44 (100%)	
*papEF*	21 (43.8%)	2 (7.7%)	1 (50%)	3 (6.8%)	ns
*papG1*	8 (16.7%)	3 (11.5%)	0 (0.0%)	8 (18.2%)	0.664
*papG2*	7 (14.6%)	4 (15.4%)	1 (50%)	2 (4.5%)	0.186
*papG3*	16 (33.3%)	2 (7.7%)	0 (0.0%)	1 (2.3%)	0.551
*sfaS*	8 (16.7%)	0 (0.0%)	0 (0.0%)	1 (2.3%)	ns
*focG*	4 (8.3%)	3 (11.5%)	0 (0.0%)	0 (0.0%)	0.048
*afa/draBC*	0 (0.0%)	3 (11.5%)	0 (0.0%)	1 (2.3%)	0.141
*bmaE*	0 (0.0%)	0 (0.0%)	0 (0.0%)	0 (0.0%)	
*gafD*	0 (0.0%)	0 (0.0%)	0 (0.0%)	0 (0.0%)	
*c2395*	24 (50%)	6 (23.1%)	0 (0.0%)	3 (6.8%)	0.069
*ppdD*	48 (100%)	26 (100%)	2 (100%)	44 (100%)	
*yadN*	30 (62.5%)	6 (23.1%)	0 (0.0%)	4 (9.1%)	0.158
*ygiL*	37 (77.1%)	16 (61.5%)	2 (100%)	32 (72.7%)	0.426

ESBL^−^, extended-spectrum beta-lactamase-non-producing strains; ESBL^+^, extended-spectrum beta-lactamase-producing strains; LVFX^S^, Levofloxacin-susceptible strains; LVFX^R^, Levofloxacin-resistant strains; ns, not significant.

**Table 8:** List of primers, expected amplicon size, and reference.

Gene	Primer (5′→3′)	Length (bp)	Reference
*fimH*	F: TGCAGAACGGATAAGCCGTGG	508	[7]
	R: GCAGTCACCTGCCCTCCGGTA		
*papEF*	F: GCAACAGCAACGCTGGTTGCATCAT	336	[61]
	R: AGAGAGAGCCACTCTTATACGGACA		
*papG1*	F: TCGTGCTCAGGTCCGGAATTT	461	[46]
	R: TGGCATCCCCCAACATTATCG		
*papG2*	F: GGGATGAGCGGGCCTTTGAT	190	[46,62]
	R: CGGGCCCCCAAGTAACTCG		
*papG3*	F: GGCCTGCAATGGATTTACCTGG	258	[46,62]
	R: CCACCAAATGACCATGCCAGAC		
*sfaS*	F: GTGGATACGACGATTACTGTG	240	[63]
	R: CCGCCAGCATTCCCTGTATTC		
*focG*	F: CAGCACAGGCAGTGGATACGA	360	[63]
	R: GAATGTCGCCTGCCCATTGCT		
*afa/draBC*	F: GGCAGAGGGCCGGCAACAGGC	559	[63]
	R: CCCGTAACGCGCCAGCATCTC		
*bmaE*	F: ATGGCGCTAACTTGCCATGCTG	507	[63]
	R: AGGGGGACATATAGCCCCCTTC		
*gafD*	F: TGTTGGACCGTCTCAGGGCTC	952	[63]
	R: CTCCCGGAACTCGCTGTTACT		
*c2395*	F: CAAAGAGCGCAGGCAGAATCC	295	[31]
	R: CCGCTGTCGCAATCTTCACAC		
*ppdD*	F: AAGCGCCATTGGTATTCCCGC	260	[31]
	R: GAGTCATGACGACGCTTAGCC		
*yadN*	F: TGGCAATGGCTGCTGGTACTG	423	[31]
	R: TTTTGCTGTAAACATCACCCGG		
*ygiL*	F: AAGGTGAAGTTATCGATGCACC	432	[31]
	R: TAGCCTGTGCCTGCACGTTACC		

## Data Availability

The original contributions presented in this study are included in the article. Further inquiries can be directed to the corresponding author.

## Abbreviations

The following abbreviations are used in this manuscript:

UTI	Urinary tract infection
UPEC	Uropathogenic *Escherichia coli*
PCR	Polymerase chain reaction
LVFX	Levofloxacin
ABU	Asymptomatic bacteriuria
ESBL	Extended-spectrum β-lactamase
CLSI	Clinical and Laboratory Standards Institute
CI	Confidence intervals
BTB	Bromothymol blue
